# Energy transfer and radiation damping in gold–MAPbI_3_ heterostructures

**DOI:** 10.1039/d5sc05386b

**Published:** 2025-10-27

**Authors:** Bikram Ghosh, Ajinkya Shingote, Janak Bhandari, Gregory V. Hartland

**Affiliations:** a Department of Chemistry and Biochemistry, University of Notre Dame Notre Dame IN 46556 USA ghartlan@nd.edu

## Abstract

Coupling excitonic systems to propagating surface plasmon polaritons (SPPs) can potentially extend the length scale for energy transport. However, experimental visualization of these coupled states is challenging. Here, leakage radiation microscopy was used to examine the SPP modes in lithographically fabricated gold nanostripes (AuNS) coated with a thin film of methylammonium lead iodide (MAPbI_3_). By combining real-space and momentum-resolved back focal plane imaging with wavelength-tunable excitation, dispersion relations, group velocities, propagation lengths, and, consequently, dephasing rates were extracted for the SPP modes. Compared to the bare AuNS structures, the AuNS/MAPbI_3_ hybrid systems exhibit increased SPP attenuation, attributed to a combination of resonant energy transfer from the SPPs to the perovskite and increased radiation damping from the change in dielectric environment of the nanostripes. Higher-order SPP modes were also observed for the AuNS/MAPbI_3_ system, allowing their group velocities to be determined.

## Abstract 2

Coupling excitonic systems to propagating surface plasmon polaritons (SPPs) can potentially extend the length scale for energy transport.

## Graphical abstract

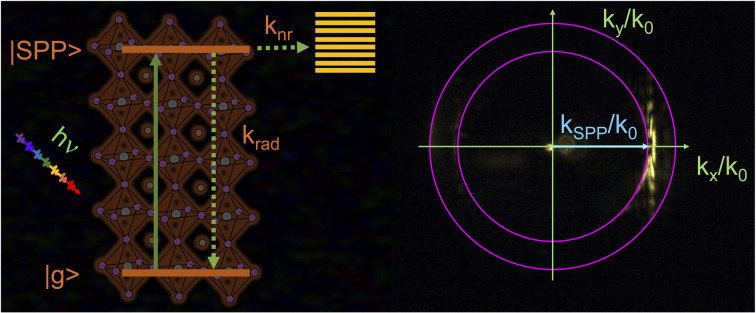

## 1. Introduction

Coupling plasmons and excitons in metal-semiconductor nanostructures can create systems that have unusual photonic properties.^1–4^ The majority of experiments have concentrated on nanoparticles that display localized surface plasmon resonances (LSPRs). LSPRs are accompanied by large field enhancements, which can lead to the creation of strongly coupled plasmon–exciton states.^1–4^ Extended metal nanostructures, such as thin films or wires, also display propagating surface plasmon polaritons (SPPs), which are electromagnetic waves bound to the metal–dielectric interfaces.^5,6^ SPPs allow the transport of optical energy over large distances along well-defined directions.^7–10^ Thus, the hybridization of excitons with SPPs is potentially useful for controlling and directing energy in photonic devices.^11,12^

The properties of coupled plasmon–exciton systems depend on the coupling strength between the two components.^2–4^ In the weak coupling regime, energy exchange between excitons and plasmons is incoherent, typically leading to increased damping.^13–15^ In contrast, the strong coupling regime is marked by coherent energy exchange that outpaces the dephasing rates, resulting in the formation of hybrid states with a characteristic Rabi splitting that directly reflects the coupling strength.^2–4,16^ Strong exciton–plasmon coupling has been experimentally demonstrated in various systems, including quantum dots and organic dye molecules coupled to metallic thin films and nanoparticles,^17–23^ and two-dimensional semiconductors interfaced with plasmonic nanostructures.^3,24–27^ These experiments have generated significant information about the energetics of plasmon–exciton coupling, however, much less is known about how such coupling affects lifetimes.^28^

In this work, leakage radiation microscopy is used to measure the lifetimes of the SPP modes in gold nanostrips (AuNSs) coupled to excitons in MAPbI_3_ perovskite thin films.^29–34^ The nanostrips were fabricated by nanolithography in order to control dimensions.^35–37^ MAPbI_3_ was chosen for these experiments for several reasons. First, hybrid organic–inorganic perovskites are interesting materials for studying plasmon–exciton coupling, due to their long charge carrier diffusion lengths.^38^ Strong plasmon–exciton coupling has also been observed for perovskites deposited on thin metal films.^39^ By combining real-space microscopy with back focal plane (BFP) imaging, we directly measure how the SPP lifetime changes when the gold nanostructures are coated with MAPbI_3_.^35–37^ These results provide new information about energy transfer from plasmons to semiconductor materials. This underpins applications such as low-threshold lasing,^40–42^ and nanoscale energy routing.^7,10^

## 2. Methods

Gold nanostripes were fabricated on #1.5 borosilicate glass substrates using a standard sequence of electron-beam lithography, metal deposition, and liftoff techniques. Electron-beam evaporation was used to deposit a 3 nm titanium layer to promote adhesion, followed by a 50 nm thick gold film. The stripes were designed to be 4 μm wide and 100 μm long. Optical images (see Fig. S1) show that the widths ranged from 3.3 to 3.7 μm, slightly different from the designed width. The MAPbI_3_ layers were deposited by spin coating a precursor solution (methylammonium iodide and PbI_2_ in DMF/DMSO mixed solvent) onto the gold nanostripes, followed by annealing (see SI for details). Different precursor concentrations were used to generate different MAPbI_3_ layer thicknesses. Absorption and emission spectra confirm the successful deposition of the MAPbI_3_, and Atomic Force Microscopy (AFM) was used to determine the thicknesses of the perovskite thin films (see Fig. S2). The AFM measurements show an average thickness of 14 ± 4 nm for the AuNS/MAPbI_3_ sample created from a 20 mM precursor solution, and 47 ± 4 nm for a 40 mM precursor solution sample (errors = standard deviations).

A diagram of the optical system used in the experiments is shown in Fig. S3. A NKT Photonics SuperK COMPACT supercontinuum white light laser was used as the excitation source. Wavelengths between 640 and 880 nm (30 nm intervals, 10 nm spectral bandpass) were selected using a series of interference filters (Thorlabs, FBHXXX-10). The laser was focused at the end of the nanostripe and polarized along the long axis to launch the SPP modes.^43,44^ Both real-space and BFP images of the leaky SPP modes of the AuNSs were recorded at each excitation wavelength.^37^ In these images, an aperture was placed at the conjugate image plane (see Fig. S3) to block the reflected laser beam, while allowing collection of scattered light from the SPP modes of the nanostripe.^35–37^ Fig. S4 presents real-space images of SPP propagation in bare AuNSs. The intensity of the scattered light decreases with distance due to attenuation from resistive heating (electron–hole pair generation due to SPP dephasing) and radiation damping, as previously reported for similar nanostructures.^35–37^ The propagation lengths for the leaky SPP modes were obtained from the real-space images by integrating the intensity over the width of the nanostripe, and fitted to a single exponential function *I*(*z*) = *I*_0_ e^−*z*/*L*_SPP_^ where *L*_SPP_ represents the SPP propagation length, see Fig. S4.^35–37^ A distinct trend of increasing SPP propagation length with increasing excitation wavelength (*i.e.*, decreasing photon energy) is observed. This behavior reflects the wavelength-dependent resistive heating losses in gold, which decrease at longer wavelengths due to reduced interband transitions, as well as weaker field confinement, which reduces energy dissipation into the metal.^34,37^

Finite element simulations of the leaky SPP modes were performed with COMSOL MultiPhysics (version 5.3a), using the Mode Analysis study in a two-dimensional electromagnetic waves, frequency domain calculation.^9^ In this model, the nanostripes were treated as rectangular structures with dimensions of 50 nm in height and 3 μm in width. A 3 nm titanium adhesion layer was incorporated between the gold and the glass substrate to match the experimental setup. For the coated structures, the MAPbI_3_ was assumed to completely cover the AuNS and the substrate. The dielectric constant data for Au was taken from ref. 45, and the dielectric constant data for MAPbI_3_ was taken from ref. 46. These simulations yield the effective index for the SPP mode *n*_eff_ − *iα*/*k*_0_, where the real part gives the SPP wavevector *k*_SPP_ = *n*_eff_*k*_0_, and the imaginary part gives the propagation length *L*_SPP_ = 1/2*α*.^9,29,30,32–36^ A 3 μm AuNS width was used to match the experimental propagation lengths and dispersion curves, see SI for details. This is slightly less than the optically measured AuNS widths, and indicates that the simple two-dimensional rectangle model does not completely capture the properties of the nanostripes.

## 3. Result and discussion

**Fig. 1:**
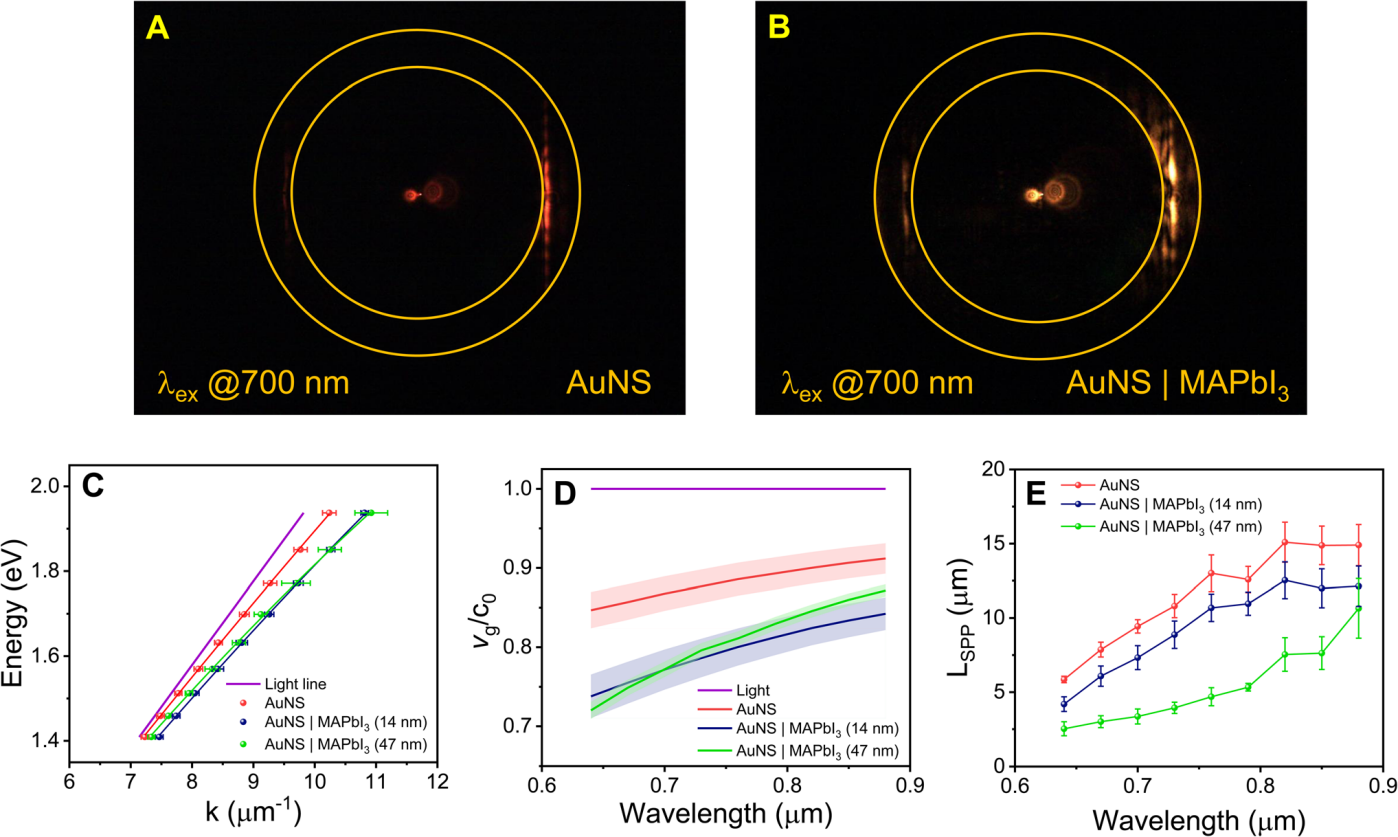
Back focal plane images of (A) a AuNS and (B) a AuNS coated with a 14 nm MAPbI_3_ thin film, both excited at 700 nm. The tangential line near the inner circle corresponds to the 1st-order leaky SPP mode wavevector. (C) Dispersion curves for the 1st-order leaky modes of bare AuNS and AuNS/MAPbI_3_ with different MAPbI_3_ thicknesses (14 and 47 nm), along with the light line for vacuum (*ω* = *c*_0_*k*_0_). (D) SPP group velocities derived from the slopes of the dispersion curves in panel (C), normalized to the speed of light in vacuum (*c*_0_). (E) SPP propagation lengths for bare AuNS and the AuNS/MAPbI_3_ samples as a function of excitation wavelength. Error bars correspond to 95% confidence limits obtained by averaging data from at least 8 nanostripes.

Fig. 1(A) displays a BFP image of the leaky SPP mode for a bare AuNS excited at 700 nm. The two circles in the image correspond to the condition for total internal reflection (*k*/*k*_0_ = 1) and the maximum wavevector that can be collected with our optical system (*k*/*k*_0_ = NA).^29–32^ The bright vertical line corresponds to the wavevector of the 1st-order leaky SPP mode for the nanostripe.^29–32,35,36^ A momentum matching diagram for coupling the leaky SPP mode to photons in the glass substrate is presented in Fig. S5 of the SI. Fig. 1(B) shows a corresponding BFP image of AuNS coated with a 14 nm MAPbI_3_ thin film. Notably, in the presence of MAPbI_3_, the SPP wavevector shifts to higher values. This is due to modification of the local dielectric environment of the nanostripe. Fig. 1(C) shows dispersion curves (frequency *versus* wavevector) of the 1st-order leaky SPP modes for the bare AuNS, and coated AuNS with 14 nm and 47 nm thick MAPbI_3_ layers. The lines are fits to the data using a quadratic function (see Fig. S6). The group velocities were subsequently determined from the derivatives (*v*_g_ = ∂*ω*/∂*k*) of these fits, and are plotted in Fig. 1(D). The average propagation lengths from eight separate nanostripes for bare and MAPbI_3_ coated AuNS are plotted *versus* wavelength in Fig. 1(E). The addition of the MAPbI_3_ layer produces a consistent decrease in the SPP propagation length across the entire spectral range investigated, and the effect is significantly stronger for the 47 nm thick MAPbI_3_ layer sample. Note that control experiments with non-absorbing thin films, such as polyvinyl alcohol or spin coating the precursor solution without PbI_2_, do not produce large changes in the leaky SPP modes (see Fig. S7).^37^ This observed reduction in *L*_SPP_ indicates that the MAPbI_3_ layer creates additional loss channels for the leaky SPP mode. Possible processes include energy transfer from the SPP modes to MAPbI_3_, as well as changes in radiation damping due to modification in the local electromagnetic environment.^37^

**Fig. 2:**
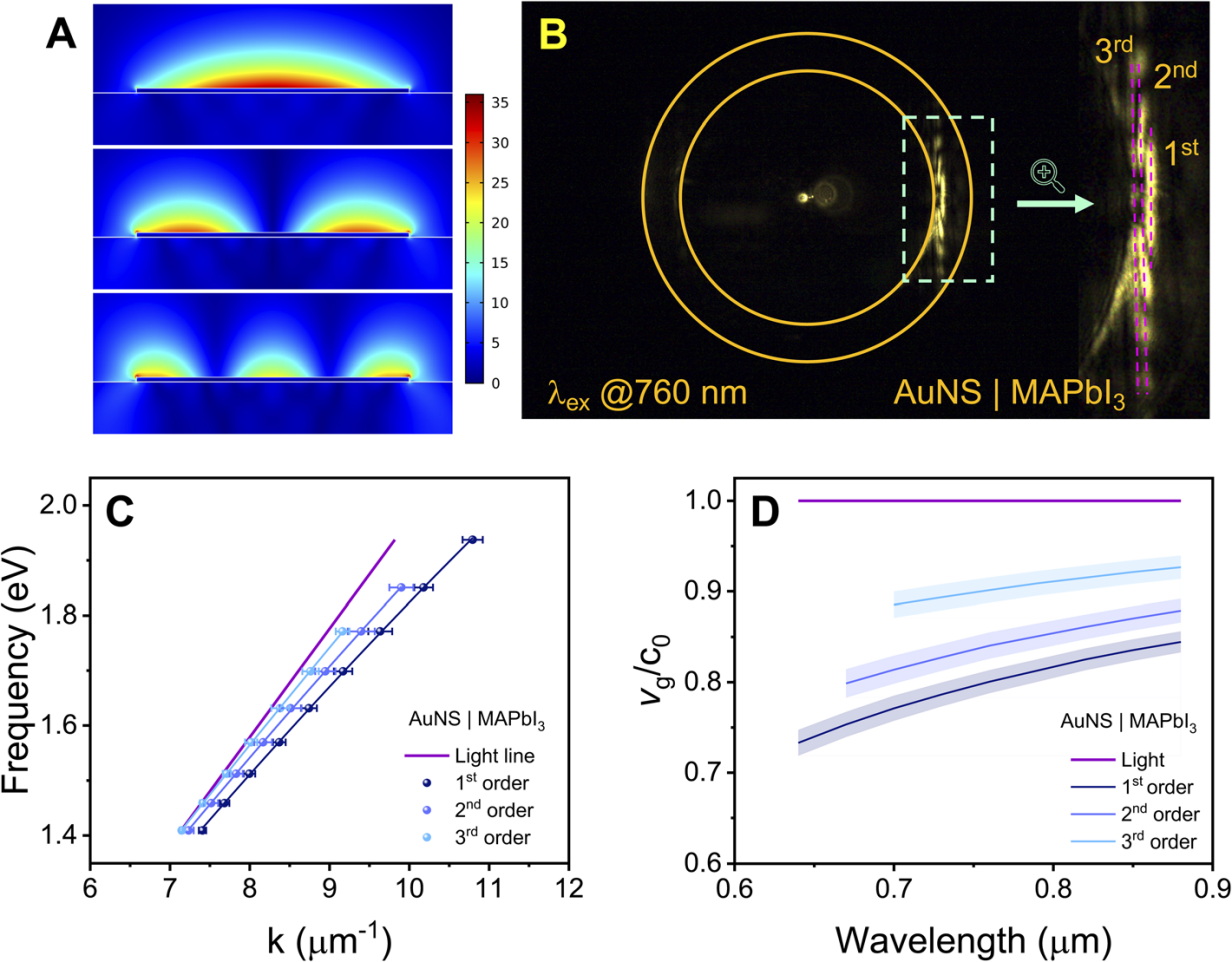
(A) Plots of the normalized electric field from finite element simulations for the 1st, 2nd and 3rd order leaky modes of a 3 μm wide AuNS. In these plots air is on the top and glass is on the bottom. (B) Back focal plane image of a AuNS coated with a 14 nm MAPbI_3_ layer at an excitation wavelength of 760 nm where multiple order leaky modes can be clearly seen. (C) Dispersion curves for the different order leaky modes in the 14 nm AuNS/MAPbI_3_ heterostructures. (D) Group velocity profiles for the different leaky SPP modes in the 14 nm AuNS/MAPbI_3_ heterostructures, normalized to the speed of light in vacuum. Error bars correspond to 95% confidence limits.

It is important to note that metal nanostripes can support multiple leaky modes.^32,36,47^ Field plots of the different order leaky modes for a 3 μm wide bare AuNS generated using finite element simulations are presented in Fig. 2(A). For the bare stripes, only the 1st order leaky mode is observed in the experiments, however, higher order modes can be seen for the coated nanostripes. Fig. 2(B) shows a BFP image for a AuNS coated with a 14 nm MAPbI_3_ thin film recorded at an excitation wavelength of 760 nm. The 1st, 2nd, and 3rd order leaky SPP modes are labeled in the image. The emergence of multiple leaky SPP modes in the AuNS/MAPbI_3_ system is attributed to the high dielectric constant of the MAPbI_3_ layer, which increases the refractive index surrounding the metal nanostripe and consequently increases the SPP wavevector, moving it away from the light line. This makes the higher order modes easier to see in the BFP images. The dispersion curves for the different order leaky modes of the 14 nm thick MAPbI_3_ layer system are presented in Fig. 2(C), and the corresponding group velocities are plotted in Fig. 2(D). The wavevectors for the higher order modes are closer to the light line and, consequently, these modes are more “light like” and have larger group velocities.^47^

The combination of the group velocity and propagation length measurements allows the SPP lifetime to be determined by *T*_1_ = *L*_SPP_/*v*_g_.^48^ This analysis is straightforward for the bare AuNS, where only one leaky mode is observed. However, it is complicated for the MAPbI_3_ coated nanostripes, which display multiple leaky modes with different group velocities. To explore the properties of the higher order leaky modes, the propagation lengths were calculated using COMSOL. Calculated dispersion curves and plots of *L*_SPP_*versus* wavelength for bare AuNS are presented in Fig. S9 of the SI. The calculations show that the higher order leaky modes have much shorter propagation lengths,^47^ which implies that the real space images yield information about the propagation length of the 1st order leaky mode (the others are quickly damped and, consequently, not observed). Thus, in the following analysis the group velocity for the 1st order leaky mode is used to determine the SPP lifetimes from the propagation length data.

**Fig. 3:**
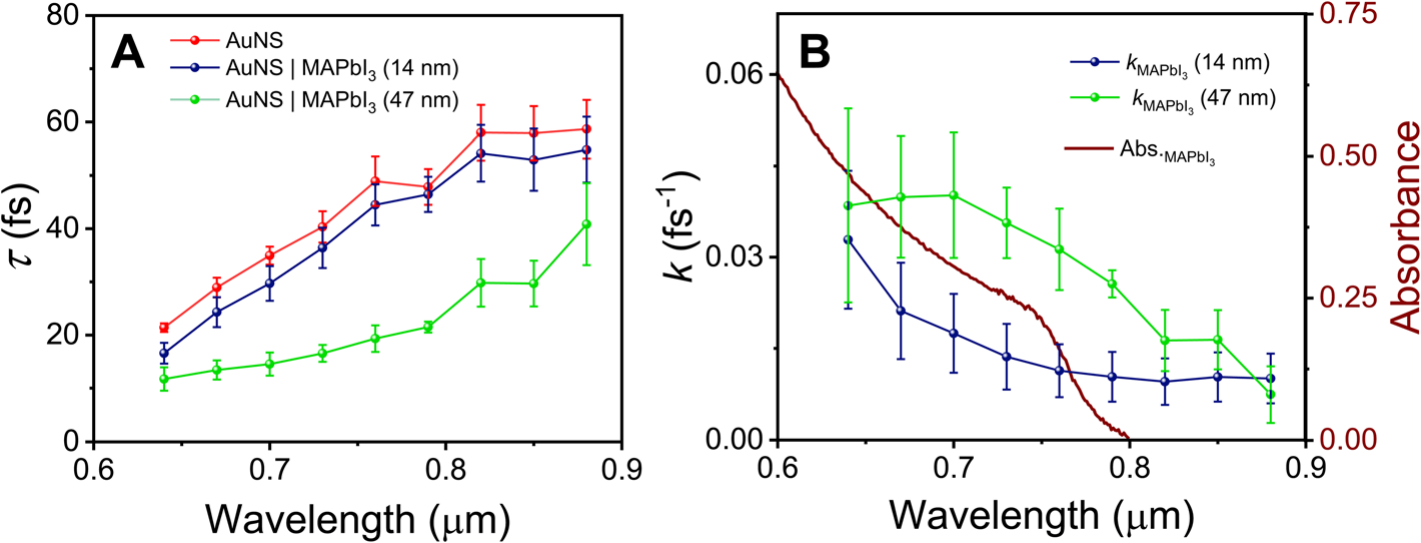
(A) SPP lifetime *versus* wavelength for bare AuNS and AuNS coated with MAPbI_3_. (B) Rate constant for SPP decay (*k*_MAPbI_3__) induced by MAPbI_3_. The red curve shows the steady-state absorption spectra of a MAPbI_3_ film prepared from a concentrated precursor solution (0.4 M). Note that the MAPbI_3_ thin films used in the SPP experiments were prepared from diluted precursor solutions: 20 mM or 40 mM for the 14 nm or 47 nm thick films, respectively. The concentrated film was used here for improved clarity. The error bars correspond to 95% confidence limits.

Fig. 3(A) shows a plot of the SPP lifetime *versus* wavelength for the bare and MAPbI_3_ coated AuNS. For the bare AuNS the SPP lifetimes range from *ca.* 20 fs at short wavelengths (*λ* < 650 nm), to just over 50 fs at long wavelengths (*λ* > 820 nm). MAPbI_3_ deposition causes a decrease in lifetime, consistent with the reduced propagation lengths observed in Fig. 1(E). The form of the change in lifetime with wavelength is different to the change in propagation length, because the group velocity is also affected by MAPbI_3_ coating. The lifetime information allows us to calculate an effective rate constant for SPP decay induced by MAPbI_3_ by *k*_MAPbI_3__ = 1/*T*_1,AuNS/MAPbI_3__ − 1/*T*_1,AuNS_, where *T*_1,AuNS_ and *T*_1,AuNS/MAPbI_3__ are the SPP lifetimes for the bare and MAPbI_3_ coated nanostripes, respectively.^37^Fig. 3(B) shows a plot of *k*_MAPbI_3__*versus* wavelength, along with a superimposed plot of the absorption spectrum of MAPbI_3_. The data shows that *k*_MAPbI_3__ is much larger for the thicker MAPbI_3_ film (as expected), and that the effect is larger above the MAPbI_3_ bandgap.

The increase in *k*_MAPbI_3__ at wavelengths above the MAPbI_3_ bandgap implies nonradiative energy transfer from the AuNS SPPs to MAPbI_3_. However, there are other potential contributions to increased SPP damping in coated nanostructures that must be considered. These include: increased damping from surface roughness introduced by the perovskite layer, chemical interface damping, and/or enhanced radiative damping due to the change in the dielectric environment of the nanostripes.^36,37^ Effects from surface roughness and chemical interface damping are not strongly wavelength dependent,^13,36,49^ and therefore do not explain the *k*_MAPbI_3__ data in Fig. 3(B). However, recent studies of dye coated Au nanostructures show that radiation damping can be wavelength dependent.^37^ Thus, to determine if the decrease in SPP lifetime is from energy transfer or radiation damping, the finite element simulations were extended to Au nanostripes coated in a thin MAPbI_3_ layer that covers both the nanostripe and the substrate. The relative contributions from radiation damping and energy transfer were determined from the dissipated powers for the different decay channels. Specifically, the dissipated power from radiation damping was calculated by integrating the time-averaged Poynting vector 
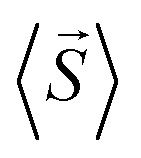
 over a circle that encloses the nanostripe: 
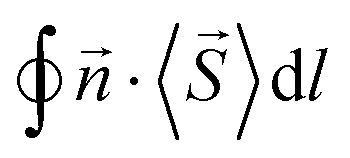
 where 
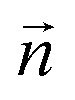
 is the outward normal unit vector. The power dissipated by energy transfer was calculated from resistive heating in the MAPbI_3_ layer: ∫∫*Q*_rh_d*A* where *Q*_rh_ is the resistive losses and the integral is over the MAPbI_3_ domain. Resistive heating in the Au was calculated in a similar way, see SI for details.^34^

Using the film thicknesses determined from the AFM measurements in the finite element simulations with the dielectric constants of bulk MAPbI_3_, yields propagation lengths that much shorter than the experimental measurements. This implies that either the film thicknesses at the nanostripes are less than that measured in the AFM experiments, and/or that the deposited film is porous. To account for the uncertainty in thickness/porosity of the film, the coated nanostripes were modelled in two different ways. In model (i) the film was assumed to have the same dielectric constants as bulk MAPbI_3_,^46^ and the thickness was adjusted to approximately match the measured propagation length at 0.85 μm. In model (ii) a porous film composed of air and MAPbI_3_ with the same thickness as the that determined by the AFM measurements was used, and the dielectric constants were calculated using an effective medium approach.^50^ The volume fraction of the film was then adjusted to match the measured propagation length at 0.85 μm. Only the 1st order leaky modes were considered in the simulations. The optimal parameters for the 14 nm thick MAPbI_3_ layer sample are a thickness of 8 nm for model (i), and a volume fraction of 0.4 for model (ii). For the 47 nm thick MAPbI_3_ layer sample the optimal parameters are 14 nm for model (i), and a volume fraction of 0.25 for model (ii). The two models make similar predictions for the relative contributions from radiation damping and resistive heating, with model (ii) providing a slightly better fit to the data, see Fig. S11.

**Fig. 4:**
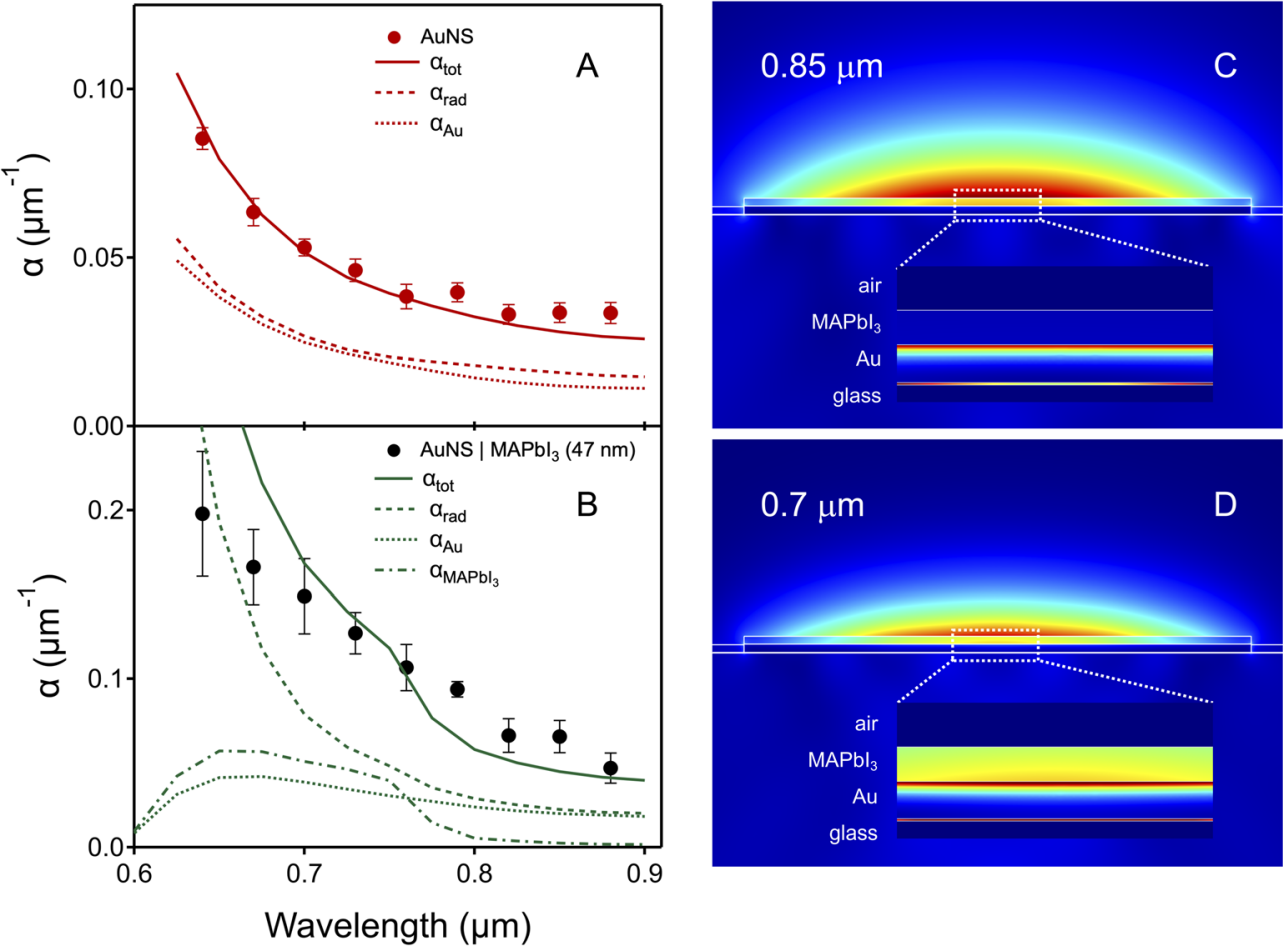
Attenuation constant *versus* wavelength for (A) bare AuNS, and (B) AuNS coated with 47 nm MAPbI_3_. Points are experimental data and lines are the simulation results: the solid lines show the total calculated attenuation constant, and the dashed, dotted and dash-dotted lines are the contributions from radiation damping, resistive heating in Au and resistive heating in MAPbI_3_, respectively. The effective medium model was used the MAPbI_3_ layer. Note the different *y*-axis scale for panels (A) and (B). (C) and (D) Normalized electric field for the coated AuNSs at 0.85 μm and 0.7 μm (below and above the MAPbI_3_ bandgap). The inserts show plots of the power dissipated by resistive heating. The thin line between the Au and glass is resistive heating in the Ti wetting layer.

The results from the finite element simulations are collected in Fig. 4. Fig. 4(A) shows calculated attenuation constants for 3 μm wide bare gold nanostripes as a function of wavelength. The dashed and dotted lines are the contributions from radiation damping and resistive heating in Au, respectively. The calculations are in good agreement with the experimental data for this choice of the nanostripe width, and show that radiation damping and resistive heating have similar magnitudes. Fig. 4(B) presents calculations for gold nanostripes coated with a 47 nm thick MAPbI_3_ layer, using the effective medium model (model (ii)). The calculations show that the contribution from resistive heating in Au is similar for the coated and uncoated nanostripes. However, there is a significant increase in SPP attenuation from both radiation damping and energy transfer to the MAPbI_3_ layer for the coated structures. Both these effects depend on wavelength. Just above the MAPbI_3_ band-edge attenuation due to energy transfer (*α*_MAPbI_3__) and radiation damping (*α*_rad_) are similar in magnitude. However, at short wavelengths (*λ* < 0.7 μm) radiation damping dominates. Note that attenuation due to resistive heating in both the Au and the MAPbI_3_ decrease at shorter wavelengths. This is attributed to a reduction in the electric field inside the nanostructures due to the strong radiation damping.

The energy transfer component is visualized in Fig. 4(C) and (D), which show plots of the normalized electric field for the coated nanostripes at 0.85 μm (below the MAPbI_3_ bandgap) and 0.7 μm (above the MAPbI_3_ bandgap) for the effective medium model. The inserts show the power dissipated by resistive heating in the different layers. Note that the magnitude of the resistive heating in the MAPbI_3_ layer is much larger at 0.7 μm compared to 0.85 μm. Within our COMSOL model this is energy transfer to the MAPbI_3_ layer.

The analysis in Fig. 4 is the main conclusion from this study. Coating the gold nanostripes with MAPbI_3_ changes the dielectric constant environment around the nanostripes, causing an increase in radiation damping. The SPP field also penetrates the MAPbI_3_ layer, which causes significant absorption in the MAPbI_3_ layer when the excitation frequency is above the MAPbI_3_ bandgap. This can be seen in the plots of the attenuation due to resistive heating in MAPbI_3_ in Fig. 4(B), as well as in the image plots of power dissipated by resistive heating in Fig. 4(C) and (D). As was observed in our previous study, the calculations show that the radiation damping effects become more important at shorter wavelengths.^37^ Note that the calculations do not exactly reproduce the form of the attenuation constant *versus* wavelength data for the coated structures. This could be due to differences in the shape of the structures compared to the idealized shape used in the simulations, or to differences in the dielectric constant of the MAPbI_3_ layer compared to the tabulated dielectric constants in ref. 46. In particular, the reported dielectric constants of MAPbI_3_ differ for measurements performed by different groups,^46,51^ and are also sensitive to effects such as humidity.^52^ However, even though the simulations do not quantitively match the experimental measurements, they provide a consistent picture of the physics of the system, that is, coating plasmonic structures with a layer of an absorbing material causes increased attenuation from both energy transfer and radiation damping.

## 4. Conclusions

Leakage radiation microscopy has been used to examine energy transfer between propagating SPPs in Au nanostripes and perovskite thin films. The results show that coating the Au nanostripes with MAPbI_3_ significantly reduces the propagation length of the leaky SPP modes. The lifetime measurements in this paper imply time-constants for SPP decay induced by MAPbI_3_ are on the order of 20–30 fs for the thicker MAPbI_3_ coated samples. Comparison to finite elements simulations reveal that the increased attenuation/reduced lifetime arises from a combination of increased radiation damping as well as energy transfer from the SPP modes to the MAPbI_3_. This is consistent with our previous study of dye coated Au nanostripes,^37^ and appears to be a general feature for plasmonic nanostructures coupled to semiconductor or molecular systems. Specifically, coating metal nanostructures with a different material changes their dielectric environment, causing changes in the amount of radiation damping. The increase in radiation damping for the leaky SPP mode is similar to the attenuation from energy transfer to the semiconductor/molecular system and, thus, cannot be neglected in analysis of measurements.

The change in dielectric environment of the Au nanostripes by coating with MAPbI_3_ also allows the observation of higher order leaky modes in the back-focal plane images. These modes are heavily damped, which means that it is not possible to measure their propagation lengths in our experiments. However, we were able to determine the group velocities of the different modes by measuring dispersion curves. The group velocities are larger for the higher order leaky modes, indicating that these modes are more “light like”.^47^

## Author contributions

BG designed experiments, wrote the paper, prepared samples, constructed optical system and collected and analyzed data; AS prepared samples, constructed optical system, collected and analyzed data, helped with writing; JB constructed optical system, performed nanofabrication and collected and analyzed data; GVH proposed experiments, helped with data analysis and simulations, helped write the paper.

## Conflicts of interest

There are no conflicts to declare.

## Supplementary Material

SC-016-D5SC05386B-s001

## Data Availability

Data is available by request. Supplementary information: details of the fabrication and synthesis methods used to produce MAPbI_3_ coated Au nanostripes; description of the optical system used for leakage radiation microscopy measurements; description of finite element simulations for coated and uncoated nanostripes. See DOI: https://doi.org/10.1039/d5sc05386b.
