# Supplementary material for: Energy transfer and radiation damping in gold–MAPbI3 heterostructures
Source: Chem Sci. 2025 Oct 27;16(48):23012–8. doi: 10.1039/d5sc05386b (PMC12577014; doi:10.1039/d5sc05386b)
Supplement: SC-016-D5SC05386B-s001 [file SC-016-D5SC05386B-s001.pdf]

Supplementary Information (SI) for Chemical Science.
This journal is © The Royal Society of Chemistry 2025

Supporting Information for:

Energy Transfer and Radiation Damping in Gold–$MAPbI_3$ Heterostructures.

Bikram Ghosh, Ajinkya Shingote, Janak Bhandari, and Gregory V. Hartland*

Department of Chemistry and Biochemistry, University of Notre Dame, Notre Dame, IN 46556, USA

Contents:

1. **Fabrication of Bare and $MAPbI_3$ Coated Gold Nanostripes: S2**
2. **Characterization of $MAPbI_3$ Thin Films: S3**
3. **Real-Space and Back Focal Plane Imaging of Propagating SPPs: S5**
4. **Finite Element Simulations: S10**

* Corresponding Author: e-mail ghartlan@nd.edu

1. Fabrication of Bare and $MAPbI_3$ Coated Gold Nanostripes:

Gold nanostripes were fabricated on #1.5 borosilicate glass substrates using a standard sequence of electron-beam lithography, metal deposition, and liftoff techniques. The stripes were designed to be 4 µm wide and 100 µm long. During deposition, a 3 nm titanium layer was first applied to promote adhesion, followed by a 50 nm gold film, both deposited via electron-beam evaporation. Figure S1 shows optical images of the fabricated nanostripes. The widths ranged from 3.3 to 3.7 µm, slightly different from the designed width.

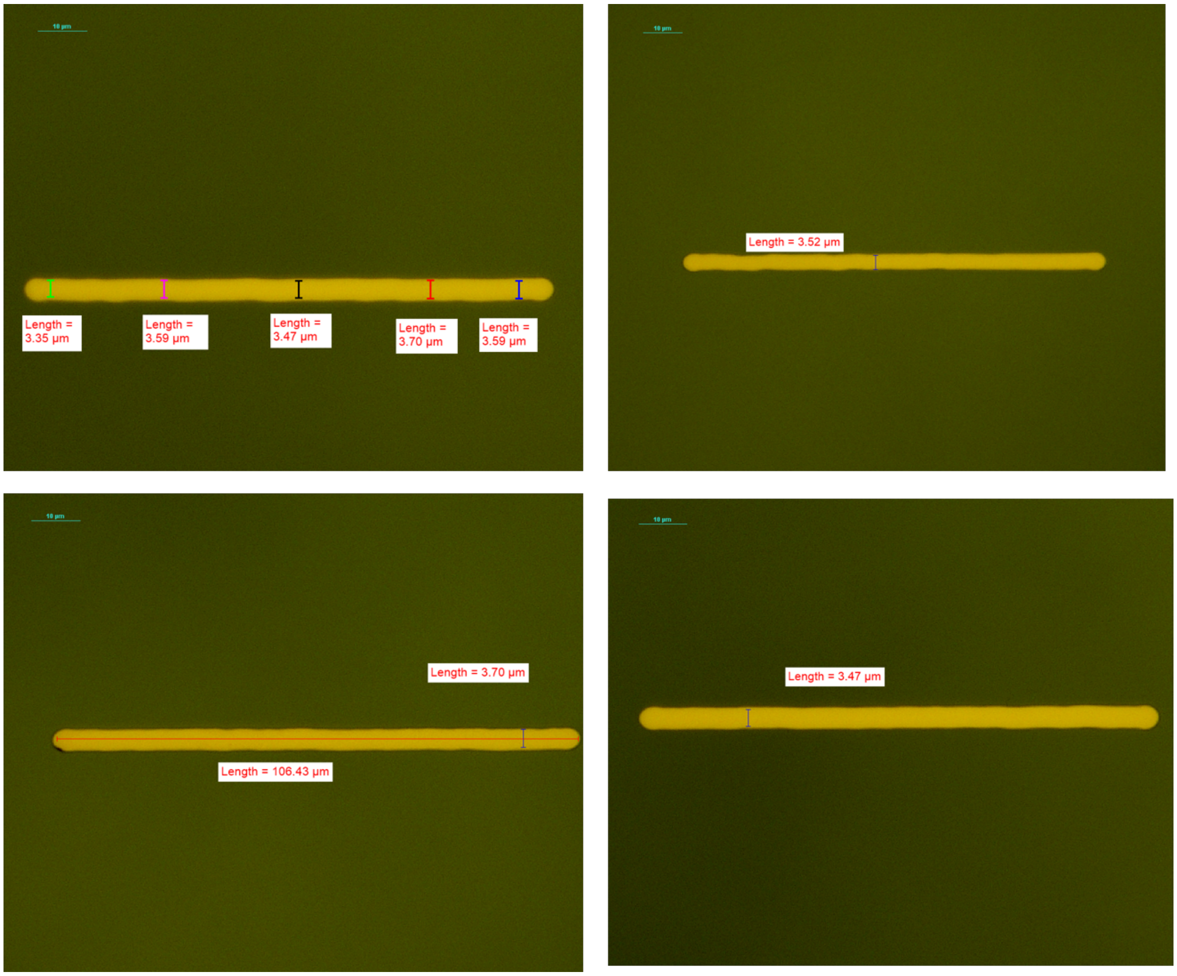

Figure S1. Transmitted light images of lithographically fabricated gold nanostrips. A noticeable variation in nanostripe width is observed both across different nanostripes, as well as along the length of individual nanostripes, reflecting inherent non-uniformities in the fabrication process.

The gold nanostripe substrates were plasma cleaned for 10 minutes to remove any remaining organics materials from the nanofabrication. The substrates were then transferred to a N_2 filled glovebox for spin casting of perovskite films. Methylammonium iodide (MAI, Greatcell Solar), lead(II) iodide (PbI_2, ultra dry, 10 mesh beads, 99.999% metals basis, Alfa Aesar), dimethyl sulfoxide (DMSO, anhydrous, ≥99.9%, Sigma-Aldrich), n,n-dimethylformamide (DMF, Sigma-Aldrich, anhydrous, 99.8%), diethyl ether (inhibitor-free, ≥99.9%, Sigma-Aldrich) were used without further purification. A 1 mL of 0.4 M stock solution of the precursors was made by dissolving 0.0635 g of MAI and 0.1844 g of PbI_2 in a 9:1 DMF/DMSO mixed solvent. The solution was left to stir for 1 hour at room temperature and subsequently filtered through an inorganic membrane filter (0.2 μm pore size, G8549141, Whatman) before use. To get the desired concentration for the experiments, the stock precursor solution was diluted 10, 20 or 40 times, i.e., concentrations of 10 mM, 20 mM or 40 mM.

The AuNS/$MAPbI_3$ films were created by static drop casting of 50 μL of precursor solution and subsequently spin coating at 4000 rpm for 25 s with an angular acceleration of 1200 rpm^2. An antisolvent treatment step with diethyl ether was used, with 0.5 mL being rapidly injected after 10 seconds. The films were immediately transferred to a preheated hotplate for 1 min at 65 °C, and then transferred to a 100 °C hotplate for 2 minutes. After annealing, the films were removed from the hotplate and left to cool before use.

2. Characterization of $MAPbI_3$ Thin Films:

Figure S2(A) shows the absorption and emission spectra obtained for perovskite films produced with the 20 mM precursor solution. The steady-state absorption spectra were measured with a Varian Cary 50 bio spectrophotometer, and the photoluminescence (PL) measurements were collected using an Edinburgh FS5 spectrofluorometer. The PL measurements were done by keeping the film inside a deaerated quartz cuvette at a 45° angle to the incident beam to minimize the scattering effect from the substrate. The film shows an absorption onset at 750 nm.

The thickness of the $MAPbI_3$ thin films deposited on the glass substrates was determined using Atomic Force Microscopy (AFM). Measurements were carried out using an Oxford Instruments Jupiter XR AFM equipped with a 20 cm sample stage. A Titan 70 probe (nominal

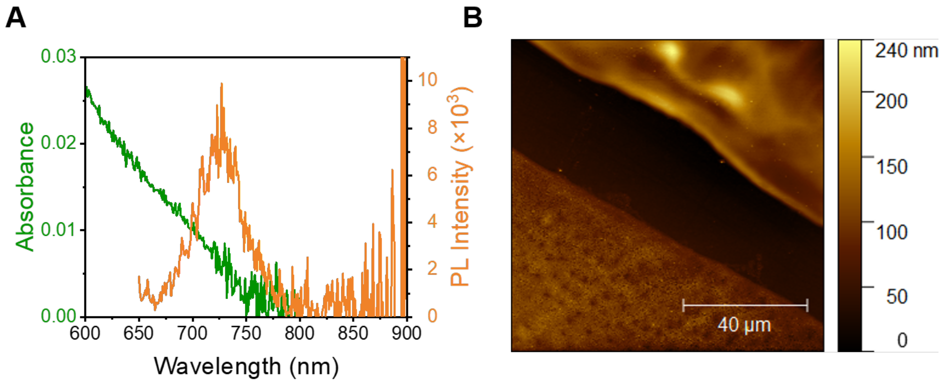

Figure S2. (A) Absorption and photoluminescence spectra of the $MAPbI_3$ thin film created with a perovskite precursor concentration of 20 mM. (B) AFM topography of a 40 mM spin-coated $MAPbI_3$ film on a glass substrate. The top-right region corresponds to residual material from the adhesive tape used to define the step edge, while the lower-left region displays the porous morphology characteristic of the perovskite film.

resonance frequency: 70 kHz; spring constant: 2 N/m) was employed in AC mode (tapping mode) to minimize surface damage and obtain high-resolution topographical data. During film preparation, glass substrates were partially masked with Kapton tape prior to spin coating. After deposition and annealing, the tape was removed to expose a clean substrate region adjacent to the perovskite film, thereby defining a well-resolved step edge for thickness determination. AFM line profiles were collected across this step, and the thickness values were extracted using the built-in analysis tools. Figure 2B presents the AFM topography of a 40 mM spin-coated $MAPbI_3$ film on a glass substrate. The top-right region corresponds to residual material from the adhesive tape used to define the step edge, while the lower-left region displays the porous morphology characteristic of the perovskite film. The AFM analysis indicates that for the 20 mM concentration, the thin films have an average thickness of 14 nm (standard deviation = 4 nm; N = 20). For the 40 mM concentration, the films exhibit an average thickness of 47 nm (standard deviation = 4 nm; N = 10). These results confirm the expected concentration-dependent increase in film thickness. The films produced at low concentration (10 mM) produced only minimal changes in the surface plasmon polariton (SPP) wavevector and propagation length compared to the bare Au nanostripes. This makes it hard to quantify the effect of the $MAPbI_3$ on the nanostripe SPP modes. Thus, only data from the 20 mM and 40 mM precursor concentration samples were analyzed.

3. Real-Space and Back Focal Plane Imaging of Propagating SPPs:

The optical experiments were conducted using an inverted microscope (Olympus IX-71). A schematic of the optical setup is presented in Figure S3. Excitation of the nanostripes was achieved with a white light supercontinuum source (NKT Photonics SuperK COMPACT). A series of interference filters were used to select the desired excitation wavelength. The laser beam was tightly focused through the glass substrate using a high-NA oil-immersion objective (Olympus UPlanFL N, 100x, NA = 1.3). Surface plasmon polaritons (SPPs) were excited by placing the end of the nanostripe at the laser focus using a motorized stage (Mad City Labs MCL-uS), where the break in symmetry allows the photons to couple to the SPPs modes. The laser polarization was aligned parallel to the stripe's long axis to optimize SPP excitation. Scattered light was collected

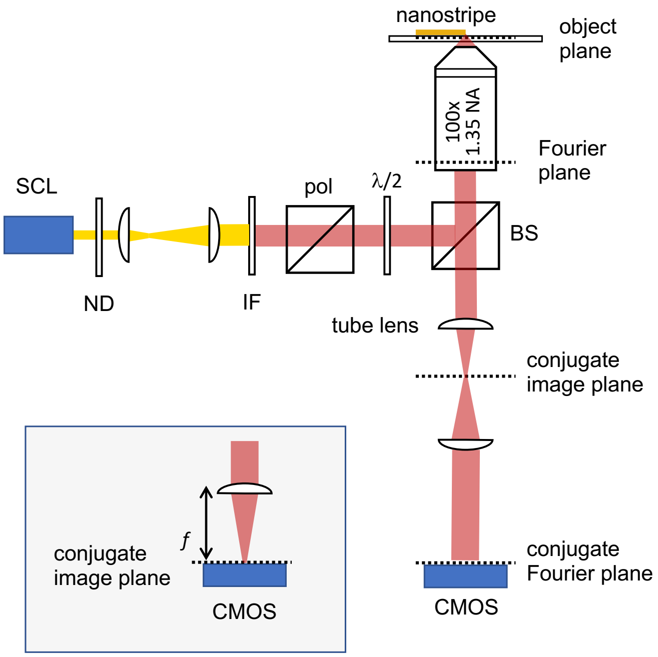

Figure S3. Diagram of the optical system for recording back focal plane (Fourier-space) and real-space images of the leaky SPP modes. The system can be converted from Fourier-space to real-space imaging by simply inserting a lens in front of the camera, as show in the insert at the bottom left of the figure. CMOS = CMOS camera; $\lambda/2$ = half-wave plate; pol = polarizer; ND = neutral density filter; IF = interference filter; SCL = supercontinuum laser.

by the same objective and directed to a CMOS camera (Basler Powerpack Microscopy Camera, Pulse 5.0 MP). A 4-f optical system was implemented to project the objective's back focal plane (BFP) onto the camera, thus enabling mapping of the scattered light's in-plane wavevector distribution onto the camera. Real-space images were obtained by inserting a lens in front of the camera – see Figure S3. To minimize background, an aperture was placed in the conjugate image plane to block directly reflected light from the substrate. Real-space images provide values for the leaky SPP mode's propagation length (L_{SPP}, see Figure S4), and the BFP images allowed extraction of the SPP wavevector (k_{SPP}, see Figure S5). Average values for L_{SPP} and k_{SPP} were determined from measurements of at least eight different nanostripes.

A momentum matching diagram for BFP imaging is given in Figure S5. Momentum matching between the leaky SPP mode and photons in the substrate occurs when $k_{SPP} = nk_0 \sin\theta \cos\varphi$, where n is the refractive index of the substrate, k_0 is the free-space wavevector, and θ and φ are the polar and azimuthal angles, respectively. Based on the coordinate system defined in Figure S5, the corresponding wavevector components in the Fourier-space image are

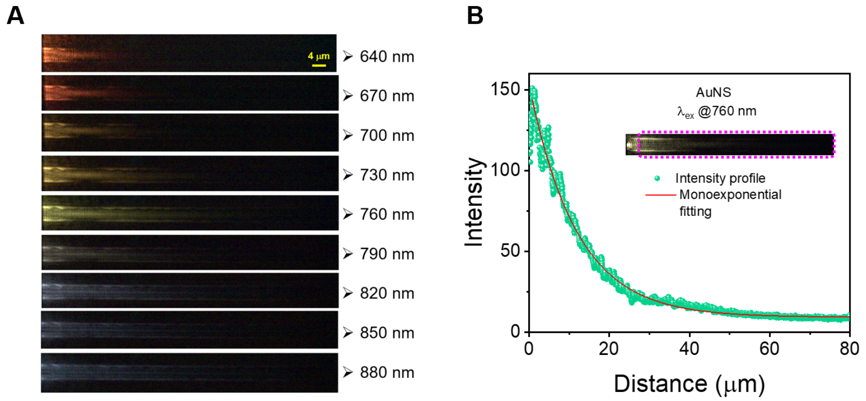

Figure S4. (A) Real-space images of leaky surface plasmon polaritons (SPPs) excited at different wavelengths. SPPs were generated by focusing a laser beam at one end of the gold nanostrip. To suppress interference from the laser, an adjustable iris was employed in the detection path to block reflected light from the substrate. (B) Intensity profile of the SPP mode at an excitation wavelength of 760 nm. The intensity was averaged over the width of the nanostripe, as indicated by the dotted area in the inset, deliberately excluding zones of strong scattered light. The resulting decay profile was fitted to a single exponential function (plus an offset) to determine the corresponding SPP propagation length.

given by $(k_x/k_0, k_y/k_0) = (k_{SPP}/k_0, k_{SPP}\tan\varphi/k_0)$ for a nanostripe oriented along the x-axis.[1] The maximum detectable wavevector is limited by the numerical aperture (NA) of the objective lens, which is indicated in the outer circle in the diagram ($k/k_0 = NA$). The inner circle corresponds to the conditions for total internal reflection $k/k_0 = \sqrt{(k_x/k_0)^2 + (k_y/k_0)^2} = 1$.

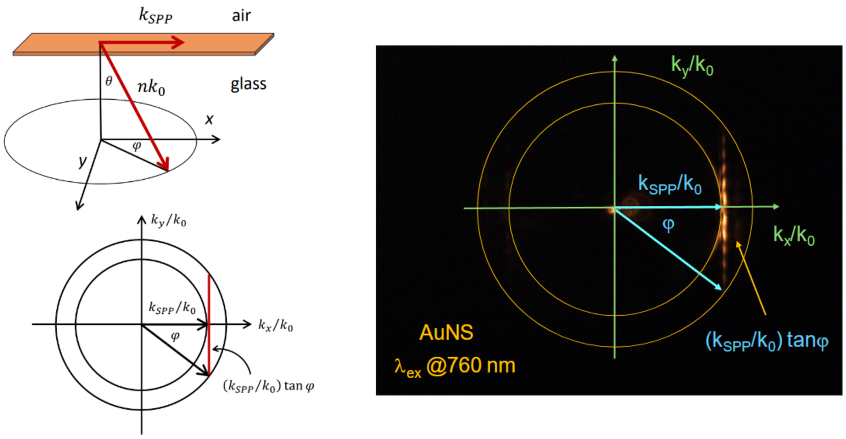

Figure S5. Left: momentum space diagram illustrating the coupling of leaky SPP modes to photons in the glass substrate. Right: example BFP image for SPPs excited at 760 nm excitation. Scattered light from the SPPs gives a sharp vertical line, which corresponds to the SPP wavevector. In this image, light directly reflected from the substrate is blocked using an aperture.

Dispersion curves are generated by measuring k_{SPP} at different excitation frequencies, and plotting frequency versus wavevector. Dispersion curves for the 1st order leaky mode of the bare nanostripes and $MAPbI_3$ coated nanostripes produced from the 20 mM concentration precursor solution are shown in Figure S6. The group velocity (v_g) of the leaky SPP mode was obtained from the dispersion plots by $v_g = \partial\omega/\partial k$. Figure S6(A) shows a fit to the frequency versus wavelength data using a straight line, and Figure S6(B) shows a fit using a quadratic function with the intercept set to zero. The quadratic function gives a better fit to the data and also is more physical, as it gives the correct limit of $\omega \rightarrow 0$ when $k \rightarrow 0$. For these reasons quadratic fits to the nanostripe dispersion curves were used to determine values for v_g in the main text.

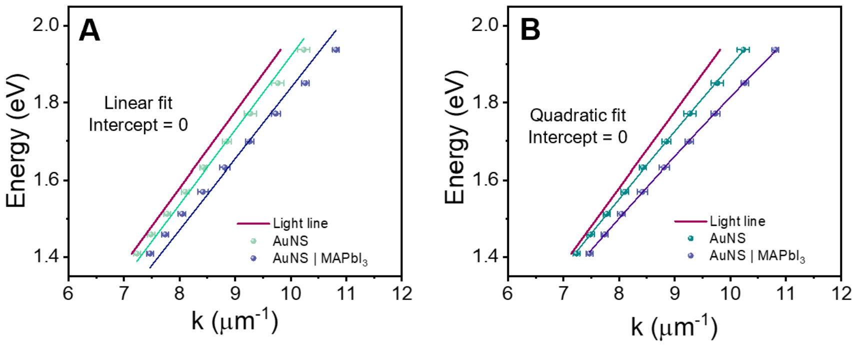

Figure S6. Dispersion curves for bare AuNS and the first-order leaky mode for the AuNS coated with 14 nm $MAPbI_3$ thin film (20 mM precursor concentration) extracted from BFP images and fitted with: (A) a linear function and (B) with a quadratic function with the intercept set to zero.

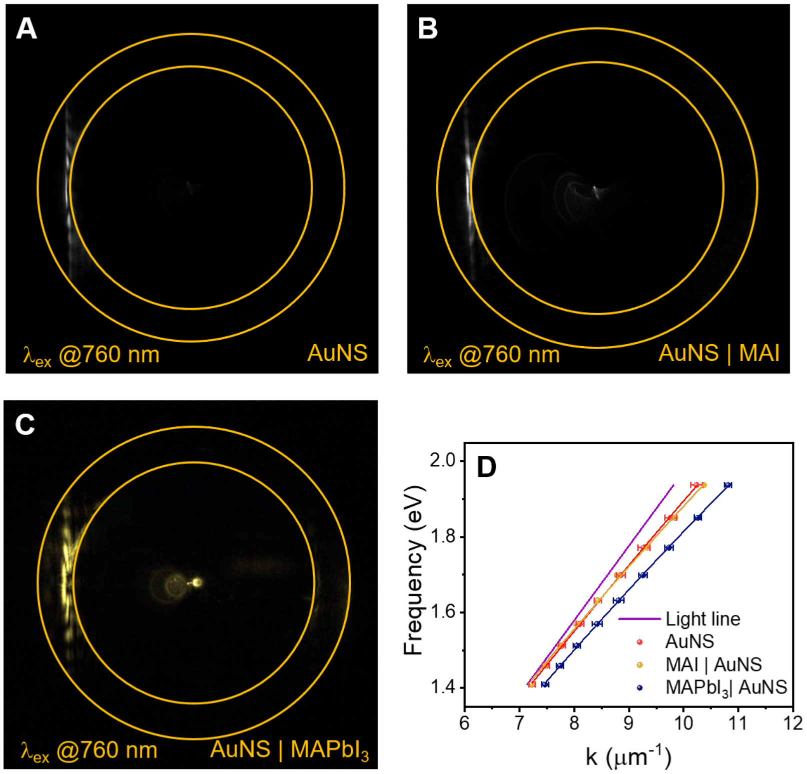

Figure S7. Fourier-space images of (A) a AuNS, (B) a AuNS coated with MAI (20 mM), and (C) a AuNS coated with $MAPbI_3$ (20 mM), all excited at 760 nm. The tangential line at the inner circle corresponds to the leaky SPP mode wavevector, (D) Dispersion curves for AuNS, MAI/AuNS, and AuNS/$MAPbI_3$, along with the light line for vacuum ($\omega = c_0 k_0$).

Several control experiments were performed. First, methylammonium iodide (MAI, 20 mM concentration) was spin coated on the Au nanostripes without PbI_2. This modifies the dielectric environment, but does not create any absorption in the 640–880 nm spectral region. Figure S7 shows BFP images and dispersion curves for the Au-MAI sample compared to bare Au and Au-$MAPbI_3$. These results show that coating with MAI has very little effect on the Au nanostripe SPP modes. In the second set of control experiments the dispersion curves and propagation lengths were measured for $MAPbI_3$ coated Au nanostripes produced using different concentration precursor solutions (10 mM, 20 mM and 40 mM). As noted above, the 10 mM precursor concentration sample does not produce significant changes in the SPP wavevector or propagation length. Changes in both the wavevector and propagation length can be observed and quantified for the 20 mM and 40 mM precursor concentration samples, and this data is presented in Figure 1 of the main text. Figure S8 shows a plot of attenuation constant versus wavelength for the 20 mM and 40 mM $MAPbI_3$ precursor concentration samples, along with a plot of the absorption spectrum of the $MAPbI_3$ thin film. As expected, there is a much larger increase in damping for the 40 mM precursor concentration sample compared to 20 mM precursor concentration sample.

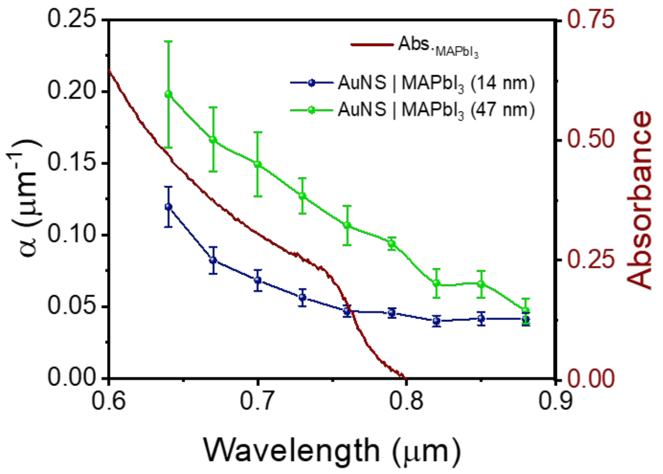

Figure S8. Attenuation constants ($\alpha = 1/2L_{SPP}$) versus wavelength for nanostripes coated with 14 nm and 47 nm thick $MAPbI_3$ thin films, prepared from precursor concentrations of 20 mM and 40 mM, respectively, and the absorption spectrum of $MAPbI_3$ prepared using a concentrated 0.4 M precursor solution.

4. Finite Element Simulations:

Simulations of the nanostripe SPP modes were performed in COMSOL Multiphysics (version 5.3a) using the Mode Analysis study in a two-dimensional Electromagnetic Waves, Frequency Domain calculation. In the model the nanostripes were treated as rectangular structures with a height of 50 nm and a width of 3 μm. A 3 nm thick titanium adhesion layer was included between the gold and the glass substrate to match the experimental setup. Optical constants for gold were taken from the refractive index data for electron-beam evaporated Au in Ref. [2], while the refractive index of the glass was set to 1.45, consistent with silica. The data in COMSOL's library of materials was used for titanium. The simulations yield the complex effective index $n_{eff} - i\,\alpha/k_0$ of the SPP modes, where the real part gives the SPP wavevector $k_{SPP} = n_{eff}k_0$, and the imaginary part is related to the propagation length by $L_{SPP} = 1/2\alpha$.[1, 3] Figure 2 of the main text shows plots of the normalized electric field for the 1st, 2nd and 3rd order leaky modes of bare nanostripes.

The calculated propagation lengths and dispersion curves are sensitive to the dimensions of the nanostripes, and their dielectric environment (that is, the thickness and refractive index of the coating). For the present sample, we find that dispersion curves and propagation lengths for the bare Au nanostripes are best reproduced by stipulating a 3 μm width – see Figure S9. This width somewhat narrower than the dimensions measured in Figure S1, and indicates that our simple two-dimensional model does not properly capture the behavior of the examined nanostripes (small deviations in shape and structural imperfections can affect the wavevectors and propagation lengths). Figure S9 also shows that the propagation lengths for the higher order leaky modes are much shorter than that for the 1st order mode. This implies that the measured propagation length in the optical experiments corresponds to the 1st order mode.

To determine whether the changes in propagation length for the coated structures arise from changes in radiation damping or resistive heating, the relative powers dissipated by the different loss mechanisms were calculated using COMSOL. Specifically, the power dissipated by radiation was calculated from the line integral of the time-averaged Poynting vector $\langle \vec{S} \rangle$ over a circle that

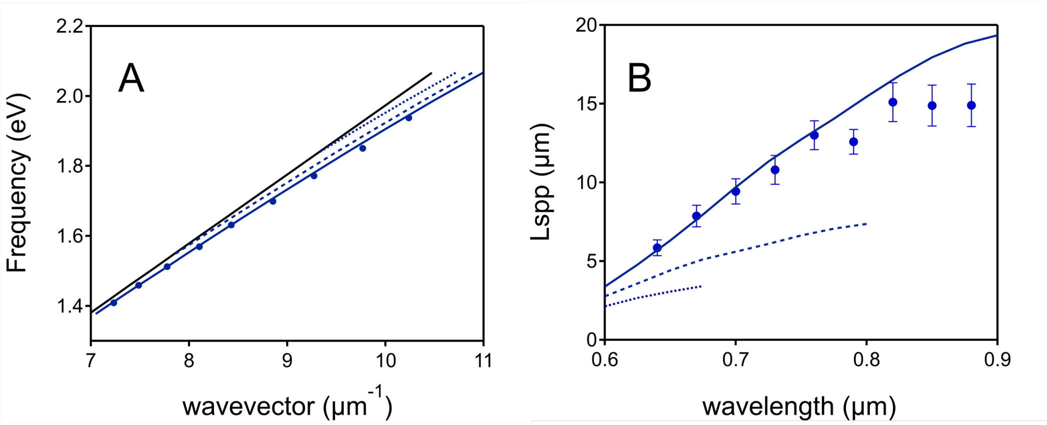

Figure S9. Calculated dispersion curves (A) and propagation lengths (B) for bare AuNS with a 3 μm width and 50 nm thickness. The solid, dashed and dotted lines show data for the 1st, 2nd and 3rd order leaky modes, and the points are the experimental data (see also Figure 2 of the main text).

encloses the nanostripe: $\oint \vec{n}.\langle\vec{S}\rangle dl$ where $\vec{n}$ is the outward normal unit vector.[1] The power dissipated by creation of excited electrons in the metal was calculated by $\iint Q_{rh} dA$ where Q_{rh} is the resistive losses in the material and the integral is over the gold and titanium domains. A similar integral was performed for the perovskite layer in the coated structures to determine the power dissipated from the SPP by energy transfer to the perovskite. The different contributions to the SPP attenuation were then calculated by $\alpha_i = \eta_i \alpha_{tot}$ where α_{tot} is the total attenuation, and η_i are the relative power losses for radiation damping (η_{rad}) and resistive heating (η_{Au} and η_{MAPbI_3}):

$$\eta_{rad} = \frac{\oint \vec{n}.\langle\vec{S}\rangle dl}{\oint \vec{n}.\langle\vec{S}\rangle dl + \iint_{metal} Q_{rh} dA + \iint_{MAPbI_3} Q_{rh} dA} \qquad (S1a)$$

$$\eta_{Au} = \frac{\iint_{metal} Q_{rh} dA}{\oint \vec{n}.\langle\vec{S}\rangle dl + \iint_{metal} Q_{rh} dA + \iint_{MAPbI_3} Q_{rh} dA} \qquad (S1b)$$

$$\eta_{MAPbI_3} = 1 - \eta_{rad} - \eta_{Au} \qquad (S1c)$$

Figure S10(A) shows the total attenuation constant for the 1st (solid line) 2nd (dashed line) and 3rd (dotted line) order leaky modes plotted versus wavelength for bare Au nanostripes. Figure S10(B) shows the contributions to the attenuation constants from radiation damping (green lines) and

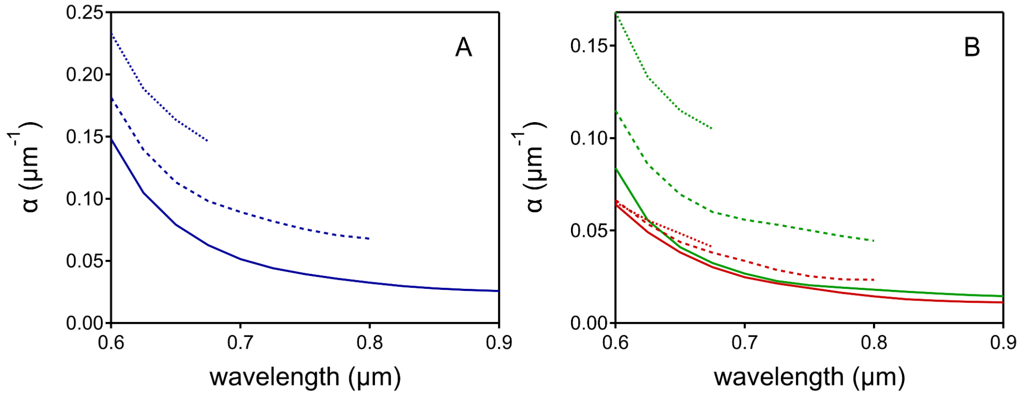

Figure S10: (A) Total attenuation constant α versus wavelength for the 1st (solid), 2nd (dashed) and 3rd (dotted) order leaky modes in bare Au NS. (B) Contributions from radiation damping (green lines) and resistive heating (red lines) to α in panel (A). The solid, dashed and dotted lines correspond to the 1st, 2nd and 3rd order leaky modes, respectively.

resistive heating in the metal (red lines). The data shows that the increased attenuation for the higher order leaky modes is primarily due to increased radiation damping.

For the simulations of the coated nanostripes a uniform layer was added to the structures. Two different models were used for the dielectric constants of the layer. In model (i) the dielectric constants of the $MAPbI_3$ layer were taken to the same as bulk material, and the thickness of the layer was adjusted to approximately match the propagation lengths at $\lambda = 0.85$ μm (below the $MAPbI_3$ bandgap). In model (ii) the coating layer was assumed to be porous, with the same thickness as that determined in the AFM measurements, and the dielectric constants were calculated using Bruggeman's effective medium theory, assuming that the film is composed of air and $MAPbI_3$:[4]

$$\varepsilon_{eff} = \frac{H_B + \sqrt{{H_B}^2 + 8\varepsilon_{\mathrm{MAPbI_3}}\varepsilon_{air}}}{4} \tag{S2}$$

where $H_B = (3x-1)\varepsilon_{\mathrm{MAPbI_3}} + (3y-1)\varepsilon_{air}$, $\varepsilon_{\mathrm{MAPbI_3}}$ is the complex dielectric function of $MAPbI_3$, and x and y are the volume fractions of $MAPbI_3$ and air, respectively. The dielectric constants of $MAPbI_3$ were taken from Ref. [5]. In this model the volume fraction of $MAPbI_3$ was adjusted to again match the measured propagation length at $\lambda = 0.85$ μm. For the 14 nm thick $MAPbI_3$ layer sample, the optimum parameters were a thickness of 8 nm for model (i), and a

volume fraction of $x = 0.4$ for model (ii). For the 47 nm thick $MAPbI_3$ layer sample, the optimum parameters were a thickness of 14 nm for model (i), and a volume fraction of $x = 0.25$ for model (ii). Figure S11 shows a comparison of the calculated and experimental attenuation constants for the two models for the 14 nm and 47 nm thick $MAPbI_3$ films. The models give similar predictions of how attenuation changes with wavelength, and the relative contributions from radiation damping, resistive heating in Au and energy transfer to $MAPbI_3$. For the 47 nm thick $MAPbI_3$ layer sample, where a clear increase in attenuation can be seen at the $MAPbI_3$ band-edge, the effective medium model gives a slightly better description of the experimental data.

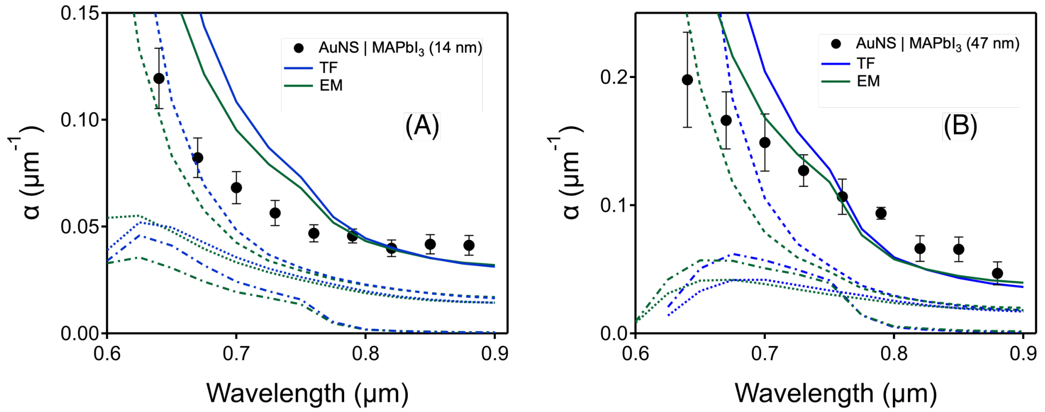

Figure S11: Attenuation constant versus wavelength for Au nanostripes coated with (A) a 14 nm thick $MAPbI_3$ layer, and (B) a 47 nm thick $MAPbI_3$ layer. Points are experimental data. The results from the thin film model (model (i)) are shown as blue lines, and those for the effective medium model (model (ii)) as green lines. The solid lines are the total attenuation constant, and the dashed, dotted and dash-dotted lines show the contributions from radiation damping, resistive heating in Au and resistive heating in $MAPbI_3$, respectively.

References:

1. Johns, P.; Beane, G.; Yu, K.; Hartland, G. V., Dynamics of surface plasmon polaritons in metal nanowires. *J. Phys. Chem. C* **2017**, *121*, 5445–5459.
2. Olmon, R. L.; Slovick, B.; Johnson, T. W.; Shelton, D.; Oh, S. H.; Boreman, G. D.; Raschke, M. B., Optical dielectric function of gold. *Phys. Rev. B* **2012**, *86*, 235147.
3. Zhang, S.; Xu, H., Optimizing Substrate-Mediated Plasmon Coupling toward High-Performance Plasmonic Nanowire Waveguides. *ACS Nano* **2012**, *6*, 8128-8135.
4. Baxter, J. B.; Schmuttenmaer, C. A., Conductivity of ZnO Nanowires, Nanoparticles, and Thin Films Using Time-Resolved Terahertz Spectroscopy. *The Journal of Physical Chemistry B* **2006**, *110*, 25229-25239.
5. Phillips, L. J.; Rashed, A. M.; Treharne, R. E.; Kay, J.; Yates, P.; Mitrovic, I. Z.; Weerakkody, A.; Hall, S.; Durose, K., Dispersion relation data for methylammonium lead triiodide perovskite deposited on a (100) silicon wafer using a two-step vapour-phase reaction process. *Data in Brief* **2015**, *5*, 926-928.
